# Salivary cell-free DNA in head and neck cancer: emerging clinical applications and future perspectives

**DOI:** 10.3389/or.2026.1885622

**Published:** 2026-07-10

**Authors:** Vaishnavi N. Patel, Aditi B. Patel, Shreya A. Lotia, Vivek M. Tanavde, Shanaya S. Patel

**Affiliations:** 1 Biological and Life Sciences, School of Arts and Sciences, Ahmedabad University, Ahmedabad, Gujarat, India; 2 Bioinformatics Institute, Agency for Science Technology and Research (A*STAR), Singapore, Singapore

**Keywords:** diagnosis, head and neck cancer, liquid biopsy, minimal residual disease, prognosis, salivary cell-free DNA, therapeutic monitoring

## Abstract

Head and neck cancer (HNC) diagnosis and surveillance still rely primarily on clinical examination, imaging, and tissue biopsy, with no established non-invasive biomarkers for routine early detection or disease monitoring. This limitation underscores the need for alternative strategies that can facilitate timely diagnosis and longitudinal assessment. Salivary cell-free DNA (scfDNA) has emerged as a promising liquid biopsy biomarker owing to its close proximity to primary HNC lesions and its suitability for non-invasive, repeatable sampling. Detection and characterization of scfDNA enable the identification of tumour-associated molecular alterations, including somatic mutations, aberrant DNA methylation, copy number alterations, viral DNA, and fragmentation signatures, providing valuable insights into tumour biology and disease dynamics. These molecular features have demonstrated considerable potential for early detection, prognostication, therapeutic monitoring, and post-treatment surveillance in head and neck cancer. However, current data are derived largely from exploratory studies with heterogeneous methodologies, small validation cohorts, and limited standardization of analytical workflows. Nevertheless, advances in sample processing, extraction methods, high-sensitivity sequencing technologies, and advanced bioinformatic approaches are enhancing assay accuracy and reproducibility. This review summarizes the current evidence on scfDNA in HNC by delineating its biological basis, evaluating the robustness and translational relevance of available clinical studies, and examining the analytical, technical, and implementation challenges that must be addressed to facilitate its integration into evidence-based clinical practice.

## Introduction

HNC is among the most common malignancies worldwide, with 2,279,479 new cases reported annually and nearly 60% of the disease burden concentrated in Asia (1). The incidence of HNC has increased over recent decades, driven by the rising prevalence of human papillomavirus (HPV)-associated oropharyngeal cancers in developed regions and the continued use of tobacco and alcohol in low- and middle-income countries. Despite advances in treatment, many patients still present with advanced-stage disease, resulting in increased loco-regional spread and poor survival outcomes. Early diagnosis remains challenging, attributed to absence of reliable, disease-specific biomarkers for screening and disease monitoring, highlighting the need for timely detection and clinical intervention.

Liquid biopsy has emerged as a promising non-invasive diagnostic approach that overcomes several limitations of conventional tissue biopsy, including sampling bias, procedural invasiveness, and the inability to capture spatial and temporal tumour heterogeneity. By enabling repeated and real-time molecular profiling through biofluids such as plasma, serum, cerebrospinal fluid, urine, and saliva, liquid biopsy offers significant potential for precision oncology (2, 3). Among these biofluids, saliva has attracted increasing interest in HNC owing to its anatomical proximity to primary tumour sites and its rich repertoire of tumour-derived biomolecules. Its simple, non-invasive collection, cost-effectiveness, and suitability for longitudinal monitoring further support its clinical utility (4–7).

**FIGURE 1:**
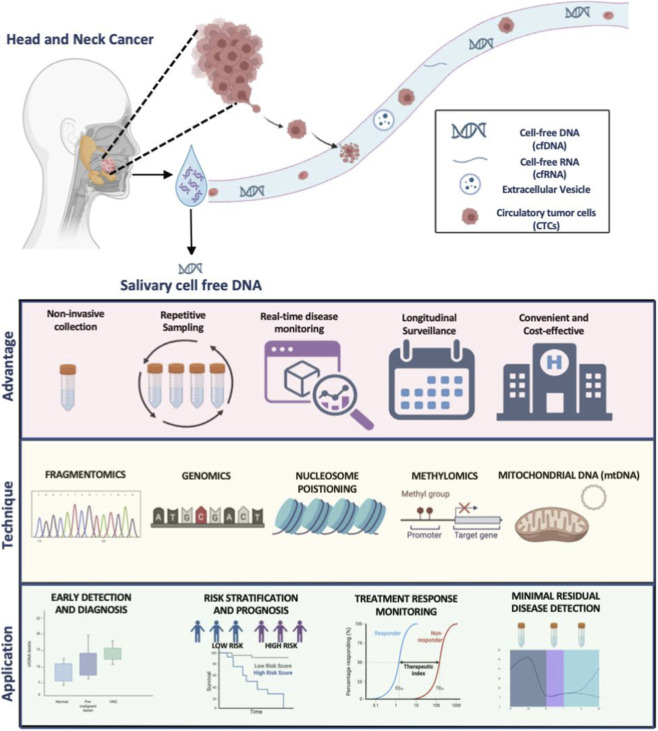
Utility of salivary cell-free DNA (scfDNA) in head and neck cancer (HNC).

Among salivary analytes, cell-free DNA (cfDNA) has emerged as a promising liquid biopsy biomarker. The tumour-derived fraction of cfDNA, termed circulating tumour DNA (ctDNA), harbours tumour-associated molecular alterations, including somatic mutations, aberrant methylation patterns, copy number changes, viral DNA, and characteristic fragmentation signatures that reflect tumour burden, clonal heterogeneity, and dynamic disease evolution (Figure 1). These molecular features have demonstrated potential clinical utility across the cancer continuum, including early detection, prognostication, tumour localization, assessment of minimal residual disease (MRD), treatment response monitoring, and recurrence surveillance (8). In HNC, saliva represents a biologically relevant biofluid because of its close proximity to primary tumours of the oral cavity and oropharynx, potentially capturing locally shed tumour DNA that may complement plasma-derived cfDNA and provide distinct insights according to tumour site and disease burden. Despite encouraging findings, the evidence supporting scfDNA remains preliminary, with studies often limited by small cohorts, heterogeneous methodologies, and insufficient external validation. Additional challenges, including low DNA yield, background contamination from non-tumour sources, variability in pre-analytical and analytical workflows, and the absence of large prospective studies, continue to impede clinical translation. This review summarizes the biological basis of scfDNA, evaluates the evidence supporting its clinical utility in HNC, and highlights the methodological and translational challenges that must be addressed to facilitate clinical adoption. Although substantial validation and standardization efforts remain necessary before routine clinical implementation, scfDNA holds immense potential as a minimally invasive biomarker capable of improving early diagnosis, therapeutic monitoring, and post-treatment surveillance in HNC, paving the way for more personalized patient management.

### Biological origin and determinants of salivary cell-free DNA

Accurate interpretation of scfDNA biomarkers requires an understanding of their biological origins and the factors influencing their abundance and composition (9–11). Unlike plasma cfDNA, which primarily reflects DNA released into the systemic circulation, scfDNA comprises nucleic acids derived from both local and systemic sources. Tumour-derived DNA enters saliva through apoptosis, necrosis, and active secretion from malignant cells, while physiological turnover of oral epithelial cells contributes substantial background host DNA (10). Immune cells within the tumour microenvironment may also release cfDNA during activation and cell death, particularly in the setting of tumour-associated inflammation. In addition, microbial DNA from the oral microbiome constitutes a significant proportion of salivary nucleic acids, necessitating analytical approaches capable of distinguishing tumour-derived sequences from non-human DNA (5–9). Recent studies have further highlighted the complexity of the salivary milieu, demonstrating that extracellular vesicles, neutrophil extracellular traps, Tumour-derived cfDNA may reach saliva through direct shedding from mucosal lesions, passive transfer from the circulation, and translocation across the mucosal barrier (10). The relative contribution of these pathways is influenced by tumour size, disease burden, vascularization, anatomical location, and proximity to salivary ducts, resulting in inter-individual variability in scfDNA composition. Exemplifying it, oral cavity and oropharyngeal tumours are more likely to release DNA directly into the oral environment, whereas laryngeal and hypopharyngeal tumours may contribute proportionally less tumour DNA to saliva. This biological heterogeneity likely contributes to the variability in diagnostic performance observed across studies and highlights the importance of interpreting scfDNA findings within the context of tumour site and clinical presentation. Recent reviews and translational studies have similarly emphasized that tumour location and disease burden are key determinants of liquid biopsy performance and should guide the selection of saliva, plasma, or combined testing strategies in HNC (12, 13).

Several biological and pre-analytical factors further influence scfDNA analysis. Endogenous salivary DNases can rapidly degrade cfDNA after collection, emphasizing the need for prompt processing or stabilization (14). Hydration status, salivary flow rate, food intake, oral hygiene practices, and circadian variation may also affect cfDNA concentration and composition (15). Importantly, inflammatory conditions such as periodontitis and oral infections have been associated with increased salivary DNA release (16), while treatment-related mucositis and radiation-induced tissue injury can elevate non-tumour host DNA levels, potentially diluting tumour-derived signals and generating false-positive findings. These considerations are particularly relevant in HNC, where poor oral health and treatment-associated mucosal toxicity are common. Recent reports have additionally emphasized the impact of pre-analytical variables, including collection devices, storage conditions, and processing delays, on cfDNA yield and integrity, highlighting the urgent need for standardized workflows to improve reproducibility across studies (17–19).

These observations indicate that scfDNA is a dynamic biomarker shaped by tumour biology, host responses, and the oral microenvironment. Understanding the determinants governing scfDNA release and composition is imperative for the accurate interpretation of biomarker studies and the development of clinically translatable assays. Collectively, the complex biological milieu of saliva and the low abundance of tumour-derived DNA underscore the need for sensitive analytical platforms and standardized workflows to enable reliable interpretation and clinical translation of scfDNA biomarkers. And inflammatory processes can influence the quantity and composition of salivary nucleic acids, thereby affecting biomarker interpretation (19).

### Analytical approaches for salivary cell-free DNA detection

The analytical platform employed for scfDNA detection is a critical determinant of assay sensitivity, specificity, throughput, and clinical applicability. Current approaches range from targeted assays assessing predefined loci to sequencing strategies capable of broader molecular profiling, each offering distinct advantages and limitations depending on the intended clinical application.

Methylation-specific PCR (MSP) and quantitative MSP (Q-MSP) provide cost-effective and highly sensitive detection of predefined CpG methylation sites but remain restricted to selected targets and may be influenced by bisulfite conversion efficiency (20–22). Droplet digital PCR (ddPCR) offers single-molecule sensitivity through reaction partitioning, enabling accurate detection of low-frequency mutations and methylation events at extremely low variant allele fractions, making it particularly suitable for the low tumour DNA abundance observed in saliva (20).

Next-generation sequencing (NGS)-based approaches, including targeted amplicon sequencing and hybrid capture panels, enable simultaneous interrogation of multiple genomic alterations but require greater DNA input and sophisticated bioinformatic pipelines to suppress sequencing artefacts (20). The incorporation of unique molecular identifiers (UMIs) and duplex sequencing has substantially improved analytical accuracy for low-input scfDNA samples (23). Whole-genome bisulfite sequencing (WGBS) enables comprehensive methylome profiling but remains costly for routine clinical application, whereas targeted bisulfite sequencing panels provide a more practical alternative (20).

Beyond mutation and methylation analysis, fragmentomic approaches based on cfDNA integrity indices and nucleosome footprinting generate tumour-agnostic molecular signatures that complement genetic analyses (18, 24). Emerging machine learning algorithms capable of integrating mutation, methylation, and fragmentation features from low-input sequencing data are expected to further improve diagnostic performance and risk stratification (23).

Importantly, pre-analytical variables including saliva collection method (unstimulated saliva versus oral rinse), collection timing, stabilization procedures, DNA extraction protocols, storage conditions, and bisulfite conversion efficiency introduce substantial inter-laboratory variability (14). Standardization of these workflows will be essential to ensure reproducibility, facilitate multicentre studies, and enable the clinical translation of scfDNA-based assays (19).

### Clinical utility of scfDNA for early detection of head and neck cancer

Timely diagnosis is critical for improving survival in HNC, yet only 17% of patients are diagnosed at stage I, with the majority presenting with advanced-stage disease due to the lack of sensitive and disease-specific biomarkers. ScfDNA has emerged as a promising liquid biopsy analyte that enables non-invasive detection of tumour-derived molecular alterations, offering new opportunities for early diagnosis and molecular characterization of HNC.

Aberrant DNA methylation is one of the most extensively investigated epigenetic biomarkers in scfDNA, encompassing both global hypomethylation associated with genomic instability and promoter hypermethylation of tumour suppressor genes that results in transcriptional silencing (18, 20–23). Frequent methylation of genes such as DAPK1, p16INK4a, and RASSF1A has been consistently reported in HNC. Importantly, accumulating evidence demonstrates strong concordance between methylation profiles in paired tumour tissue and salivary cfDNA, supporting saliva as a reliable surrogate for tumour-derived epigenetic alterations and highlighting its potential for non-invasive early detection and molecular characterization of HNC. Study by Rosas *et al.* (25) demonstrated significant concordance in promoter hypermethylation of p16, MGMT, and DAPK1 using MSP between paired tumour and saliva samples (p < 0.001) in a single-centre smaller cohort of patients (n = 30) and controls, providing preliminary evidence that salivary DNA accurately reflects tumour epigenetics. Similar observations have subsequently been reported in independent studies of nasopharyngeal carcinoma, supporting the reproducibility of these methylation markers across different populations. Nevertheless, the available evidence remains largely derived from relatively small cohorts without external validation, necessitating prospective multicentre studies before clinical implementation (26–30). Similarly, Dmitry *et al.* (31) evaluated HNC patients (n = 143) and healthy controls (n = 77) using MSP, reported a sensitivity of 94% and specificity of 87% for a methylation panel comprising RASSF1A, DAPK1, and p16. Comparable findings have been described by various independent groups, further supporting the diagnostic potential of methylation-based scfDNA assays. However, the absence of large external validation cohorts and standardized analytical workflows limits their immediate clinical applicability.

The diagnostic performance of salivary methylation profiling has been further enhanced through multi-gene panels. Liyanage et al. (32) reported diagnostic accuracies exceeding 90% using a panel comprising p16INK4a, RASSF1A, TIMP3, and PCQAP/MED15 in oral cancer patients (n = 54), oropharyngeal cancer patients (n = 34), and healthy controls (n = 60) using MSP, while Lim et al. (33) demonstrated similar performance in an independent cohort of HNC patients (n = 133) stratified by HPV status and healthy controls (n = 122) using MSP. Expanding beyond targeted approaches, Chi et al. (34) employed genome-wide methylation profiling in matched pre- and postoperative oral squamous cell carcinoma (OSCC) tissue and salivary DNA from small patient cohort (n = 13), identifying differentially methylated regions in GABRB3, IL11, INSR, NOTCH3, NTRK3, and PXN, highlighting the potential of methylome-wide analysis for biomarker discovery.

In addition to unstimulated whole saliva, oral rinse samples have emerged as a valuable source of tumour-derived DNA owing to their higher epithelial cell content, ease of collection, and improved standardization, thereby expanding the diagnostic potential of saliva-based liquid biopsy. Genome-wide methylation profiling by Guerrero-Preston et al. identified HOXA9 and NID2 as promising biomarkers through MSP, demonstrating high diagnostic accuracy in an independent cohort comprising HNC tumours (n = 179) and normal tissues, with subsequent validation in salivary rinse samples that yielded area under the curve (AUC) values of up to 0.77 (35). The diagnostic potential of NID2 was further supported by Srisuttee et al., who validated promoter hypermethylation in an independent cohort of OSCC patients (n = 43) and healthy controls (n = 50) using MSP, reinforcing its utility as a non-invasive biomarker for oral cancer detection (36). Similarly, Demokan et al. identified EDNRB and KIF1A hypermethylation in an equivalent cohort comprising of tumour tissues, pretreatment salivary rinses from HNC patients (n = 71), and healthy controls (n = 61) using MSP, demonstrating the feasibility of methylation-based detection using oral rinse specimens (37). Subsequent studies reported sensitivities of approximately 70%–80% and specificities approaching 90% for methylation panels incorporating NID2, EDNRB, and KIF1A, supporting their diagnostic potential while highlighting the need for external multicentre validation. Outlier-based methylation analysis has further improved biomarker specificity. Gaykalova et al. identified ZNF14, ZNF160, and ZNF420 as highly specific methylation markers by using Infinium HumanMethylation450 array + MSP validation through a discovery cohort of primary HNC tissues (n = 44) and normal mucosal samples (n = 25), followed by validation in an equivalent smaller cohort of HNC patients and controls, achieving 100% specificity in both tissue and salivary rinse samples (38). However, these findings remain exploratory owing to the relatively small single-centre cohort and lack of independent validation.

Additional methylation markers have also demonstrated potential for identifying premalignant lesions and malignant transformation. Hypermethylation of ECAD, TMEFF2, RARβ, and MGMT was reported by MSP in mouth rinse samples from patients (n = 34) and controls (n = 24) (39), while ZNF582 and PAX1 methylation using Q-MSP correlated with histological progression in a cohort of oral epithelial samples (n = 267) comprising oral potentially malignant disorders, OSCC, and healthy controls (40). Importantly, ZNF582 hypermethylation has subsequently been independently validated as a predictor of malignant transformation in larger cohorts, representing one of the more reproducible saliva-based methylation biomarkers.

Beyond DNA methylation, soluble CD44 (solCD44), identified by enzyme-linked immunosorbent assay (ELISA), a protein whose expression is influenced by CD44 methylation, distinguished HNC patients (n = 102) from individuals with benign conditions (n = 69) with sensitivities of 62%–70% and specificities of 75%–88% (41). Its clinical relevance has been further strengthened by a subsequent multi-institutional clinical study demonstrating that elevated salivary CD44 and total protein levels following treatment are associated with an increased risk of disease recurrence. Likewise, EDNRB hypermethylation has been investigated as a biomarker using Q-MSP for dysplasia and OSCC in high-risk individuals, with reported sensitivities ranging from 46% to 71% and specificities of 58%–72% across independent cohorts (42, 43).

Collectively, current evidence identifies DNA methylation as one of the most promising and extensively investigated scfDNA biomarkers for the non-invasive detection, molecular stratification, and early diagnosis of HNC and oral premalignant lesions. Findings from both whole saliva and oral rinse studies demonstrate encouraging diagnostic performance and biological concordance with tumour tissue. However, the available evidence is largely derived from single-centre studies with modest sample sizes, heterogeneous analytical methodologies, and limited independent external validation. Consequently, standardized collection and analytical protocols, together with large prospective multicentre studies, are essential to establish the reproducibility and clinical utility of methylation-based scfDNA assays before their routine implementation in HNC management.

Beyond epigenetic alterations, salivary cfDNA harbors tumour-specific genetic variants that provide complementary molecular information by directly capturing oncogenic driver mutations associated with HNC. Mutation profiling, therefore, represents an important component of scfDNA-based liquid biopsy, enabling molecular characterization alongside methylation analysis. Owing to its close anatomical proximity to primary mucosal lesions, saliva may be particularly informative for detecting locoregional tumour-derived mutations, especially in early-stage disease. Wang *et al.* demonstrated, in a multicentre cohort of HNC patients (n = 93) using Safe-SeqS, that scfDNA detected HPV16 DNA in approximately 75% of early-stage cases and showed complementary sensitivity to matched plasma samples, highlighting the added diagnostic value of saliva-based analysis (44). These observations are consistent with independent studies that have validated salivary HPV16 DNA as a reliable surrogate for tumour HPV status in oropharyngeal squamous cell carcinoma (OPSCC) (45, 46). Likewise, Shanmugam et al. employed ultra-sensitive targeted sequencing in a smaller cohort of OSCC patients (n = 121) and identified recurrent alterations in CASP8, PIK3CA, FAT1, CDKN2A, NOTCH1, HRAS, and TP53, with mutation profiles confirmed against matched tumour tissue, supporting their tumour origin (47). Although these findings demonstrate the ability of scfDNA to capture clinically relevant genomic alterations, current evidence remains limited by relatively small cohorts and requires prospective multicentre validation before routine clinical implementation.

Beyond its genetic and epigenetic composition, salivary cfDNA retains structural features that reflect tumour biology. Characteristic fragmentation patterns arising from altered nucleosomal organization and tumour-associated cell death have emerged as promising biomarkers for the non-invasive detection of HNC (24). Rapado-González *et al.* evaluated fragmentation indices using Quantitative PCR (ALU-based fragmentation/qPCR) in a preliminary single-centre study comprising OSCC patients (n = 19) and healthy controls (n = 15), reporting significantly elevated ALU115/ALU60 and ALU247/ALU60 integrity indices in cancer patients, with AUC values of 0.82 and 0.70, respectively (48). Nevertheless, current evidence remains exploratory, with limited cohort sizes and an absence of external validation, highlighting the need for larger prospective studies to establish fragmentation profiling as a clinically robust biomarker.

Salivary mitochondrial cell-free DNA (scf-mtDNA) has emerged as a promising complementary biomarker for HNC owing to its high copy number, enhanced stability, and association with tumour-associated metabolic reprogramming (49–51). Sayal *et al.* assessed salivary cell-free mitochondrial DNA (scf-mtDNA) expression in patients with HNC (n = 102), individuals with oral leukoplakia (n = 31), and healthy controls (n = 137). Scf-mtDNA demonstrated good discriminatory performance for HNC detection, yielding an AUC of 0.826 and a diagnostic accuracy of 80.5%, which was higher than that observed for salivary nuclear cfDNA (77.4%) (52). Collectively, these findings suggest that scf-mtDNA may serve as a valuable adjunct to nuclear cfDNA analysis for HNC detection. However, further multicentre validation and assay standardization are required to establish its clinical utility and integration into multiplex liquid biopsy platforms.

Thus, current evidence highlights scfDNA as a multifaceted liquid biopsy analyte capable of capturing complementary genetic, epigenetic, and structural alterations associated with HNC. Methylation profiling remains the most extensively investigated approach, while somatic mutation analysis, fragmentation signatures, and mitochondrial cfDNA provide additional layers of molecular information that may enhance diagnostic sensitivity and tumour characterization. Although these biomarkers have demonstrated encouraging performance across multiple studies, most evidence is derived from single-centre cohorts with heterogeneous methodologies and limited external validation. Consequently, standardized pre-analytical and analytical workflows, together with large prospective multicentre studies, are essential to establish the reproducibility and clinical utility of multi-analyte scfDNA assays. The integration of complementary molecular signatures within salivary cfDNA has the potential to enable accurate, non-invasive, and early detection of HNC, paving the way for precision diagnostic strategies and improved patient outcomes.

## Emerging role of scfDNA in prognosis and risk stratification of HNC

The persistently high mortality associated with HNC, accounting for nearly one million deaths annually worldwide, underscores the need for improved prognostic biomarkers that can refine risk stratification and guide personalized patient management (1). Integration of scfDNA into existing prognostic frameworks offers a non-invasive approach for dynamic risk assessment and longitudinal disease monitoring.

Among scfDNA-derived biomarkers, DNA methylation signatures have shown the most consistent prognostic utility. Carvalho et al. evaluated hypermethylation of a five-gene panel (CCNA1, DAPK, DCC, MINT31, and p16) in salivary rinses from 61 HNC patients, across multiple subsites using Q-MSP and demonstrated that the panel independently predicted both local recurrence (hazard ratio [HR] = 12.2; 95% confidence interval [CI] = 1.8–80.6; *p* = 0.010) and overall survival (HR = 2.8; 95% CI = 1.2–6.5; *p* = 0.016) (53). Consistent with these observations, Righini et al. demonstrated that hypermethylation of TIMP3, ECAD, p16, MGMT, DAPK, and RASSF1 in salivary samples using MSP was associated with poorer clinical outcomes, including reduced overall survival (36.5%, p < 0.015) and diminished local disease control (60.8%, p = 0.01) (54). Among these markers, TIMP3 promoter hypermethylation has emerged as a particularly promising prognostic biomarker. Rettori *et al.* reported, in a single-centre cohort, that persistent salivary TIMP3 methylation by Q-MSP independently predicted local recurrence-free survival (p = 0.025) (55). Importantly, this observation was subsequently validated by Sun *et al.* in an independent cohort of HNC patients (n = 197), in which salivary TIMP3 hypermethylation identified using Q-MSP was significantly associated with poorer recurrence-free survival in both univariate (HR = 2.61, 95% CI = 1.16–5.84) and multivariate analyses (HR = 2.51, 95% CI = 1.10–5.68) (56). Although the available evidence is encouraging, TIMP3 hypermethylation should currently be regarded as a promising investigational biomarker whose clinical value lies in its potential to complement established prognostic factors and improve risk stratification in HNC.

Alongside DNA methylation markers, tumour-specific mutations detected in scfDNA have also emerged as promising prognostic biomarkers in HNC. Ping Wu et al. analysed paired salivary and plasma-derived ctDNA from 27 HPV-negative HNC patients using targeted next-generation sequencing and reported TP53 mutation detection in 88.2% of saliva samples compared with 68.4% of plasma samples (57). TP53 mutations were associated with shorter disease-free survival and predicted relapse with a sensitivity of 62.5% and specificity of 89.5% for salivary ctDNA, and a sensitivity of 62.5% and specificity of 94.7% for plasma ctDNA. Suggesting that salivary ctDNA may provide greater sensitivity for detecting locoregional tumour-derived mutations. Collectively, these findings highlight the potential of salivary TP53 mutation analysis as a non-invasive prognostic biomarker for identifying patients at increased risk of recurrence and disease progression, while emphasizing the need for further validation in larger, prospective cohorts.

Taken together, current evidence suggests that salivary cfDNA-derived methylation signatures and tumour-specific mutations provide complementary molecular information for prognostic assessment and risk stratification in HNC. By enabling non-invasive monitoring of recurrence risk and disease progression, scfDNA-based biomarkers have the potential to complement conventional clinicopathological factors and support personalized surveillance and therapeutic decision-making. Nevertheless, standardized analytical workflows and large prospective multicentre studies are required to facilitate their translation into routine clinical practice.

### Role of salivary cell-free DNA in therapeutic monitoring and minimal residual disease detection

Despite significant advances in chemotherapy, radiotherapy, and multimodal treatment strategies, HNC continues to exhibit high recurrence rates, with approximately 50%–60% of patients experiencing disease relapse (58). As most recurrences are locoregional and occur within the first 2 years following primary treatment, accurate monitoring of therapeutic response and early detection of residual or recurrent disease are essential for improving patient outcomes and long-term survival.

Among scfDNA-derived biomarkers, aberrant DNA methylation has shown considerable potential for post-treatment monitoring. Aberrant promoter hypermethylation has been associated with 5-year overall and disease-free survival in OPSCC, highlighting its utility as a molecular marker for surveillance (59). In a cohort comprising post-treatment OPSCC patients, treatment-naïve patients, and healthy controls, Shen *et al.* demonstrated that 75% of recurrent HPV-positive patients exhibited detectable salivary HPV DNA detected using MSP + qPCR (HPV E5 DNA), while 87.5% showed at least one positive biomarker (60). Importantly, combining HPV DNA (HR E5L2–4) with hypermethylated EDNRB improved diagnostic performance (AUC = 0.88) compared with either marker alone (AUC = 0.69–0.78), suggesting that integrated viral and epigenetic scfDNA biomarkers may enhance recurrence surveillance. However, the translational significance of these findings is limited by methodological heterogeneity, restricted methylation panel coverage, and the relatively small exploratory cohort. Validation using standardized analytical workflows in larger independent multicentre studies will be essential to confirm their clinical utility for post-treatment surveillance.

Similarly, Cui *et al.* employed whole-exome sequencing to analyse paired salivary and plasma cfDNA from 11 OSCC patients and demonstrated that salivary cfDNA mutations could reflect treatment response and detect recurrence earlier than conventional clinical assessment (61). In particular, salivary cfDNA identified single-nucleotide variants up to 4 months before clinical recurrence and showed 72.7% concordance with primary tumour DNA, outperforming plasma cfDNA (9.1%). Nevertheless, the small sample size and absence of external validation limit the broader applicability of these findings, and the study should be regarded as an exploratory proof-of-concept investigation.

The ability of scfDNA to dynamically reflect tumour burden also positions it as a promising biomarker for minimal residual disease (MRD) detection. Although MRD assessment in solid tumours remains challenging because of tumour heterogeneity and variable cfDNA shedding, advances in ultra-sensitive sequencing and digital PCR technologies have expanded the feasibility of ctDNA-based MRD detection in HNC (62–64). Within this context, methylation profiling has emerged as one of the most promising approaches for identifying residual disease following curative treatment. In a noteworthy study in OSCC patients, salivary cfDNA methylation analysis by targeted methylation sequencing identified five tumour-specific methylation markers in 70% of patients before surgery, with these signals largely disappearing following complete tumour resection (65). Persistence of at least one methylation marker 4 weeks after surgery was significantly associated with close or positive surgical margins (AUC = 0.71; p = 0.0429), achieving a sensitivity of 0.67 and specificity of 0.63. Notably, persistent ASCL1 methylation predicted recurrence 4 months before clinical detection in one patient, suggesting the potential of methylation-based surveillance for early relapse identification. However, the exploratory nature of the study, together with its limited sample size and lack of independent validation, restricts the robustness and broader applicability of the findings. Moreover, the association between ASCL1 methylation and recurrence was based on a single patient, precluding a reliable assessment of its prognostic significance. Complementing methylation-based approaches, Ferrier et al. prospectively evaluated HPV-specific ctDNA in paired saliva and plasma samples from HPV-associated HNC patients (n = 77) using ddPCR (66). Combined analysis of both biofluids achieved 93.5% sensitivity and 98.7% specificity for detecting residual disease and recurrence, with a 93% concordance between saliva and plasma ctDNA positivity. ctDNA detection declined from 91% before treatment to 8% after treatment (p < 0.00001), and persistent post-treatment ctDNA was associated with residual disease, highlighting its potential for treatment surveillance and MRD detection. While these findings are encouraging, prospective multicentre validation will be important to confirm their clinical utility.

Collectively, these studies highlight the ability of salivary cfDNA to capture dynamic molecular changes associated with therapeutic response, residual disease, and tumour recurrence in HNC. Longitudinal assessment of methylation patterns, tumour-specific mutations, and HPV-derived ctDNA offers a real-time molecular snapshot of disease evolution that could complement conventional imaging and clinical follow-up. Although further harmonization of analytical approaches and prospective validation are required, the integration of scfDNA into post-treatment surveillance strategies holds considerable promise for enabling earlier intervention and more personalized patient management.

## Discussion: current evidence and translational challenges

The studies reviewed herein collectively highlight the considerable promise of scfDNA as a minimally invasive biomarker for the management of HNC. Across the continuum of care, scfDNA has demonstrated potential utility in early detection, prognostication, therapeutic monitoring, and post-treatment surveillance through the identification of tumour-associated mutations, methylation signatures, viral DNA, and fragmentomic patterns. However, despite these encouraging findings, the current evidence base remains preliminary and insufficient to support routine clinical implementation.

A major limitation of the existing literature is the predominance of small, single-centre, exploratory studies employing heterogeneous methodologies and patient populations. Variations in tumour subsites, HPV status, disease stage, saliva collection protocols, analytical platforms, and reporting endpoints hinder direct comparisons across studies and complicate the interpretation of reported diagnostic and prognostic performance. Moreover, many biomarkers demonstrating high sensitivity and specificity have been evaluated in limited cohorts without independent validation, raising concerns regarding reproducibility and the potential for overfitting. Consequently, the distinction between promising exploratory biomarkers and clinically validated assays remains unclear.

An additional consideration is the biological and clinical context in which scfDNA testing is applied. Unlike plasma-derived cfDNA, which primarily reflects systemic tumour burden, scfDNA may better capture locally shed tumour DNA, particularly in malignancies arising within the oral cavity and oropharynx. This complementary relationship suggests that saliva and plasma should not be regarded as competing biofluids but rather as distinct sources of tumour information whose relative utility may vary according to tumour site, disease burden, and clinical objectives. Combined liquid biopsy strategies integrating both matrices may therefore provide superior diagnostic and surveillance performance compared with either approach alone.

Several pre-analytical barriers and analytical challenges continue to impede the translation of scfDNA into routine clinical practice by substantially influencing assay performance. In addition, regulatory considerations, quality-control requirements, cost-effectiveness, and integration into established clinical pathways remain largely unexplored. Addressing these issues through standardized operating procedures and multicentre collaborative efforts will be essential to ensure reproducibility and facilitate broader clinical adoption.

Taken together, the available evidence supports the biological plausibility and clinical potential of scfDNA as a liquid biopsy analyte in HNC. Nevertheless, substantial validation is still required before scfDNA-based assays can transition from investigational tools to evidence-based clinical applications. Future studies should prioritize rigorous prospective evaluation, external validation, and standardized methodological frameworks to define the settings in which scfDNA can provide meaningful improvements in patient care.

## Future directions

Future research should prioritize large prospective multicentre studies with independent validation cohorts and standardized pre-analytical and analytical workflows to establish the clinical utility of scfDNA in HNC. Dedicated evaluation of scfDNA performance in HPV-positive and HPV-negative disease is warranted, given their distinct biological characteristics and biomarker profiles. The integration of tumour-informed and tumour-agnostic approaches, together with multi-omic strategies combining mutational, methylation, fragmentomic, and viral DNA analyses, may further improve diagnostic accuracy and risk stratification. In addition, artificial intelligence and machine learning-based classifiers offer opportunities to enhance biomarker interpretation and predictive modelling from complex datasets. Finally, cost-effectiveness analyses and implementation studies will be essential to determine the feasibility, value, and impact of incorporating scfDNA-based assays into routine clinical practice.

Thus, scfDNA is a promising liquid biopsy analyte with immense potential in various clinical applications of HNC patient management. Further prospective validation and harmonization of analytical workflows are required before scfDNA-based assays can be incorporated into routine clinical practice. With continued research and technological refinement, scfDNA may become a valuable component of precision oncology strategies for patients with head and neck cancer.
